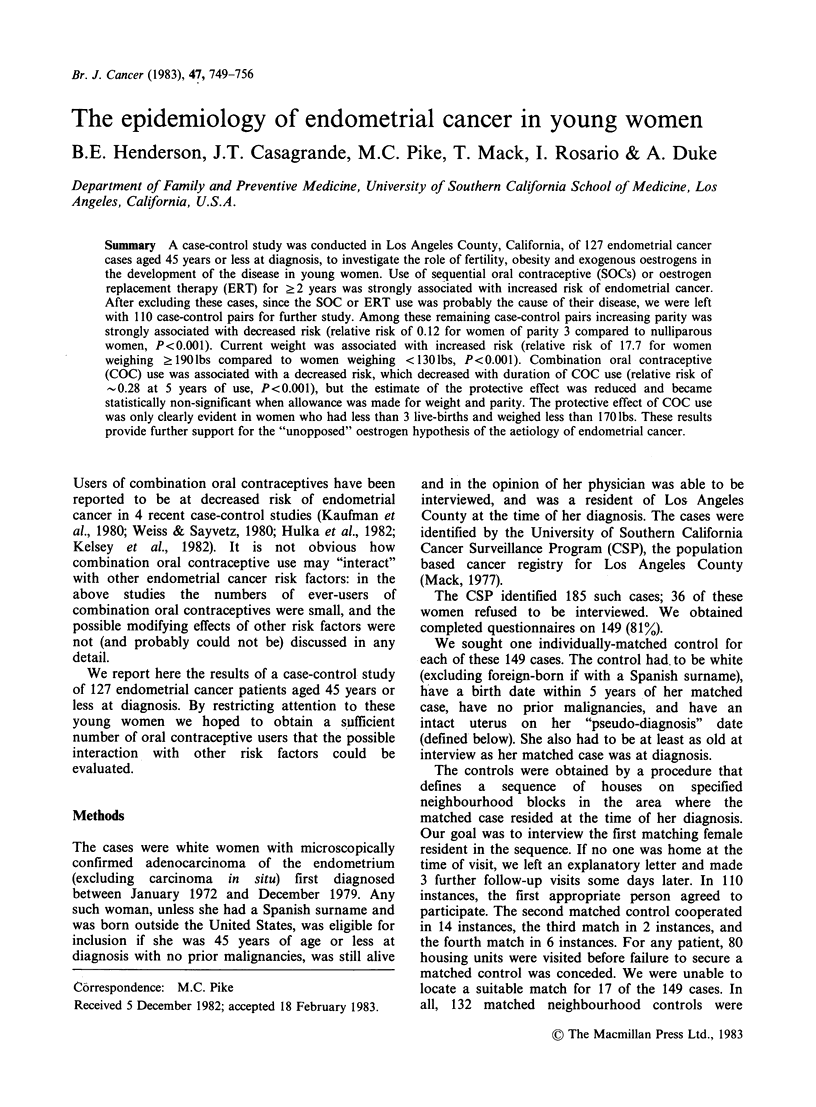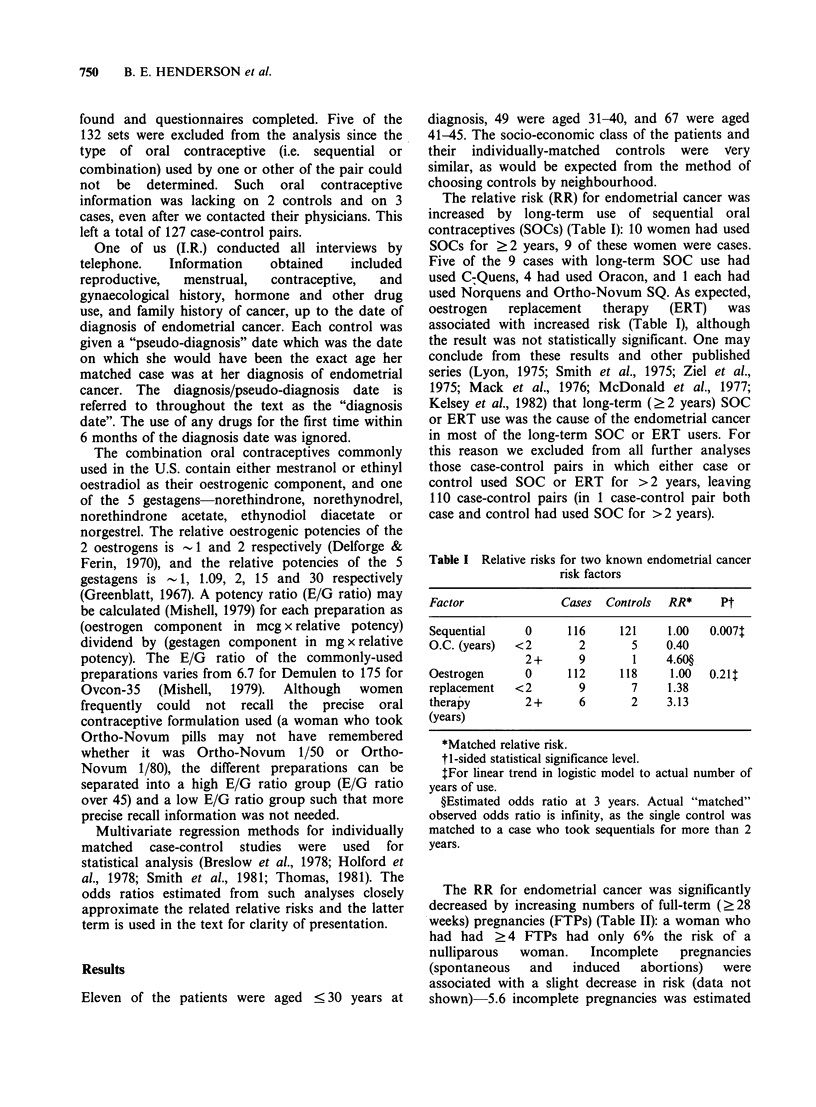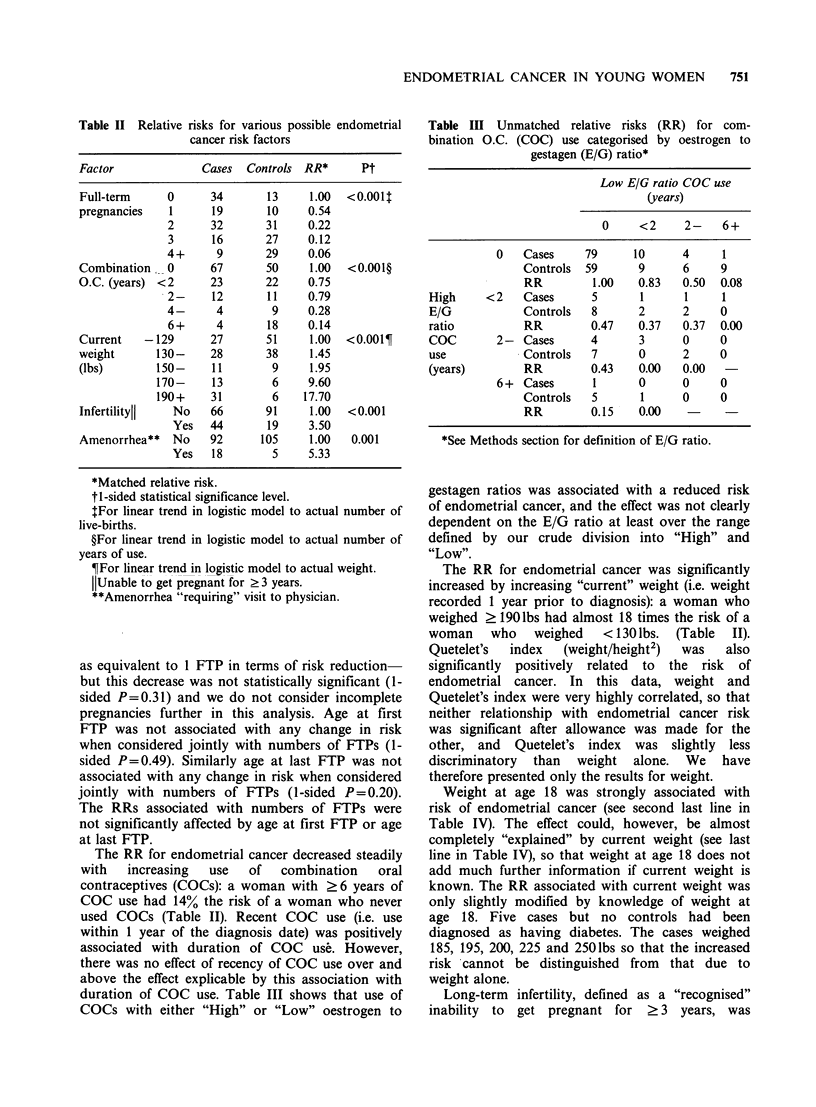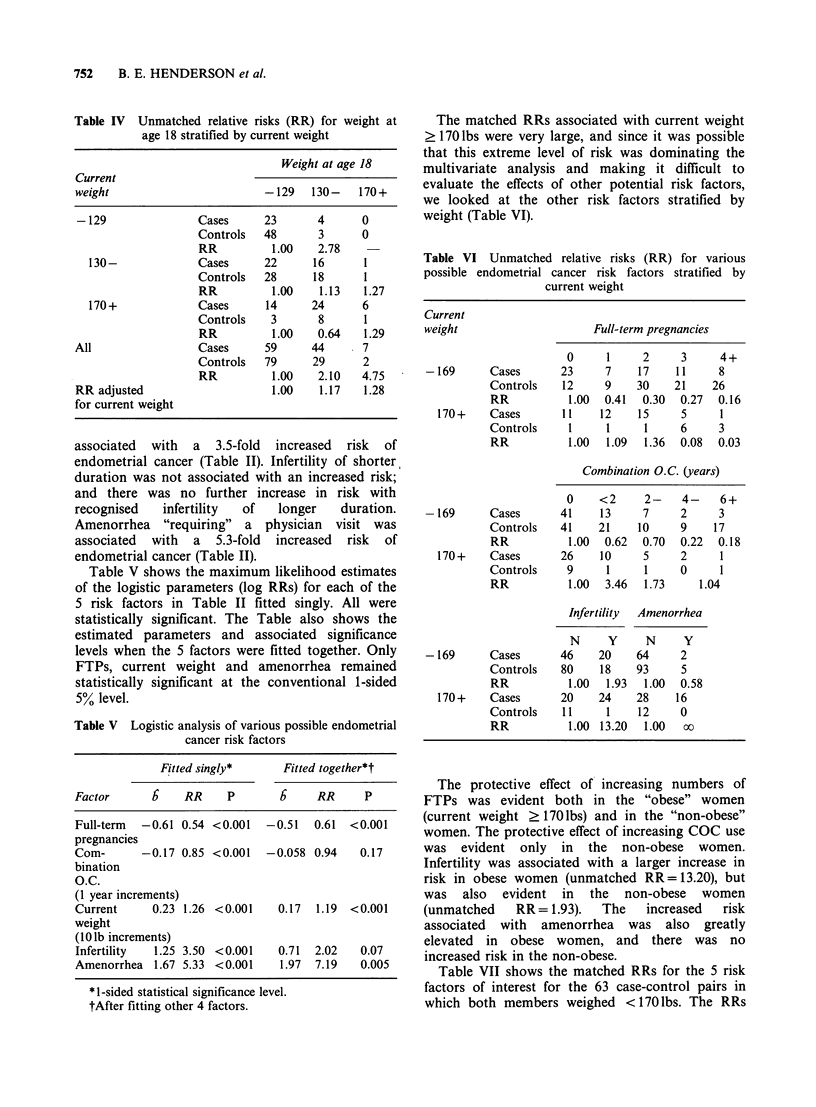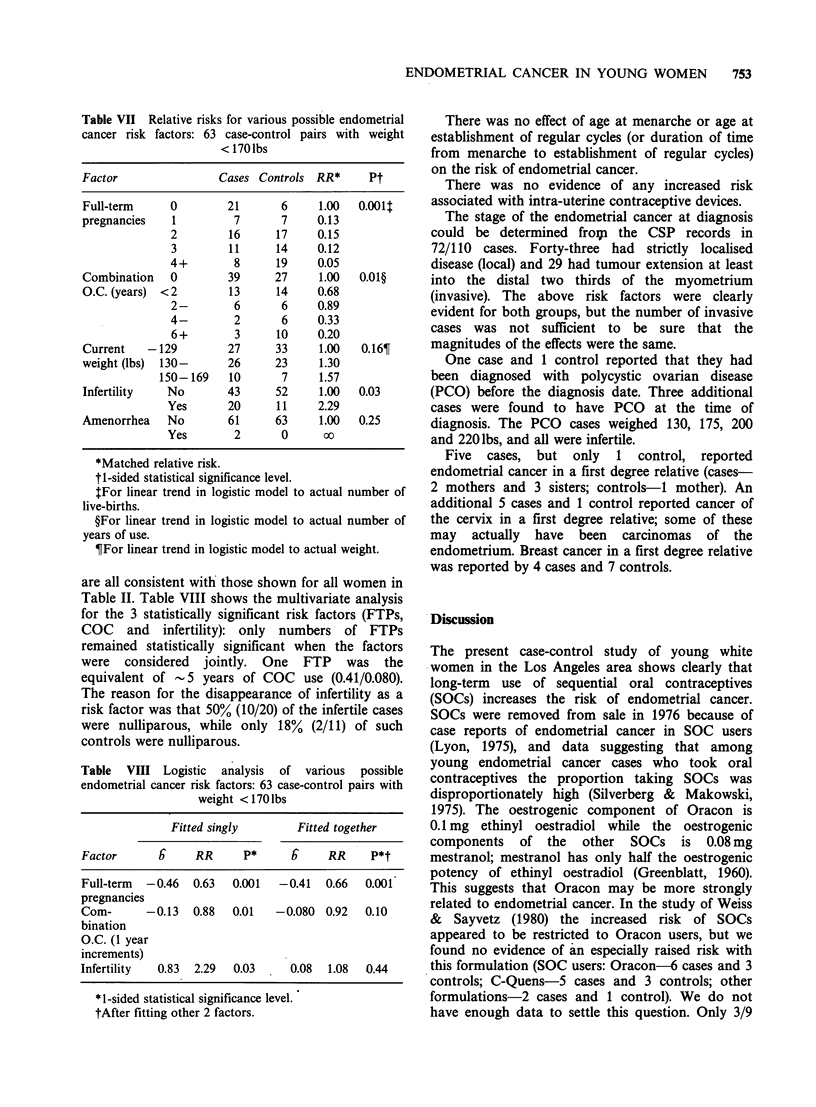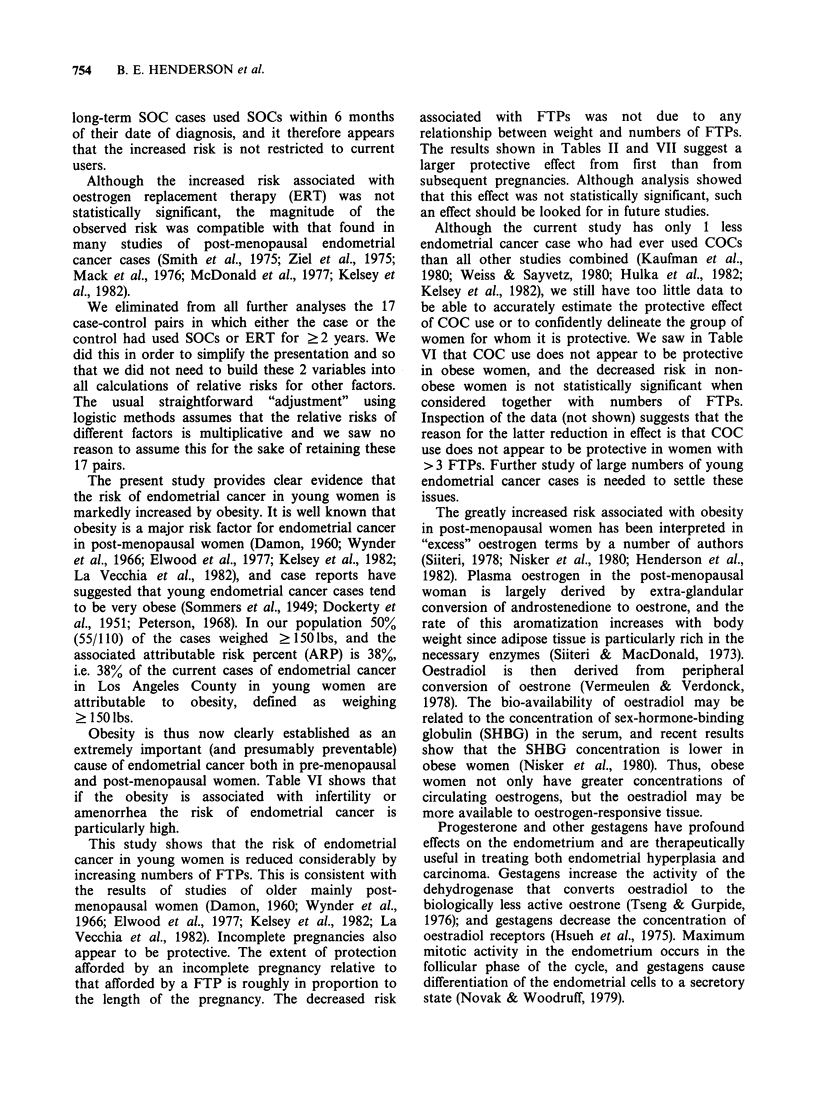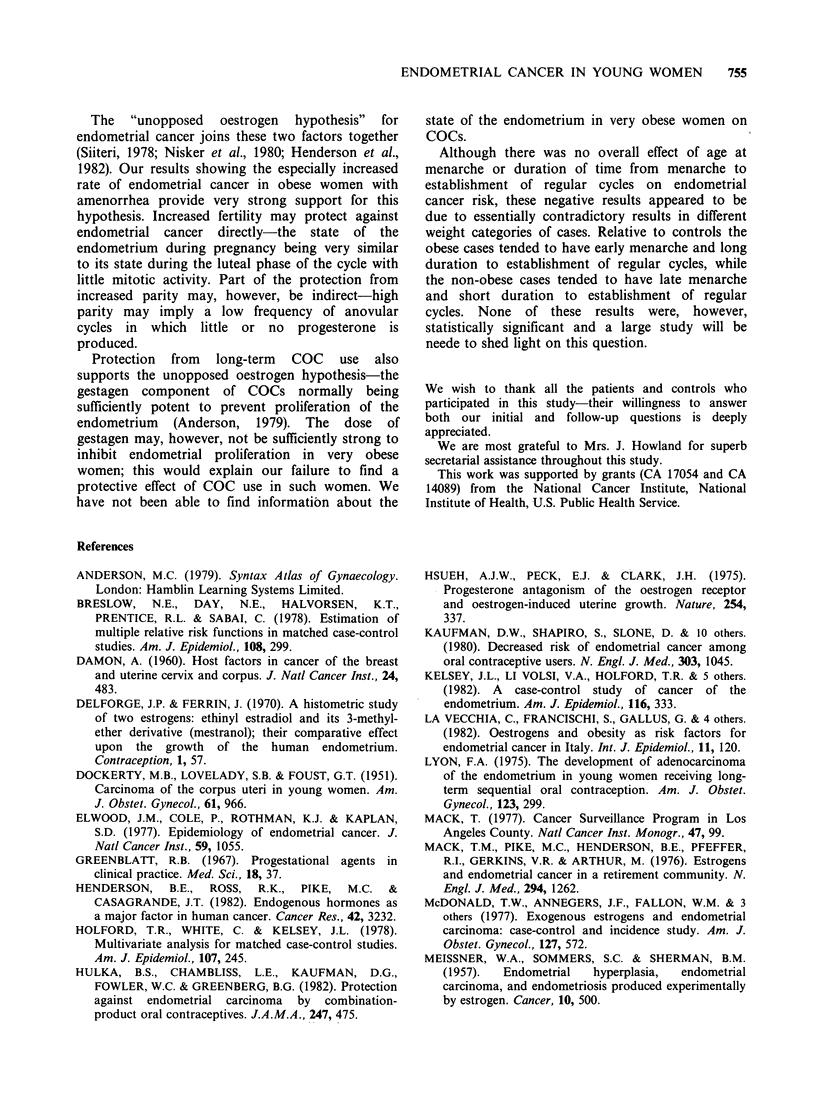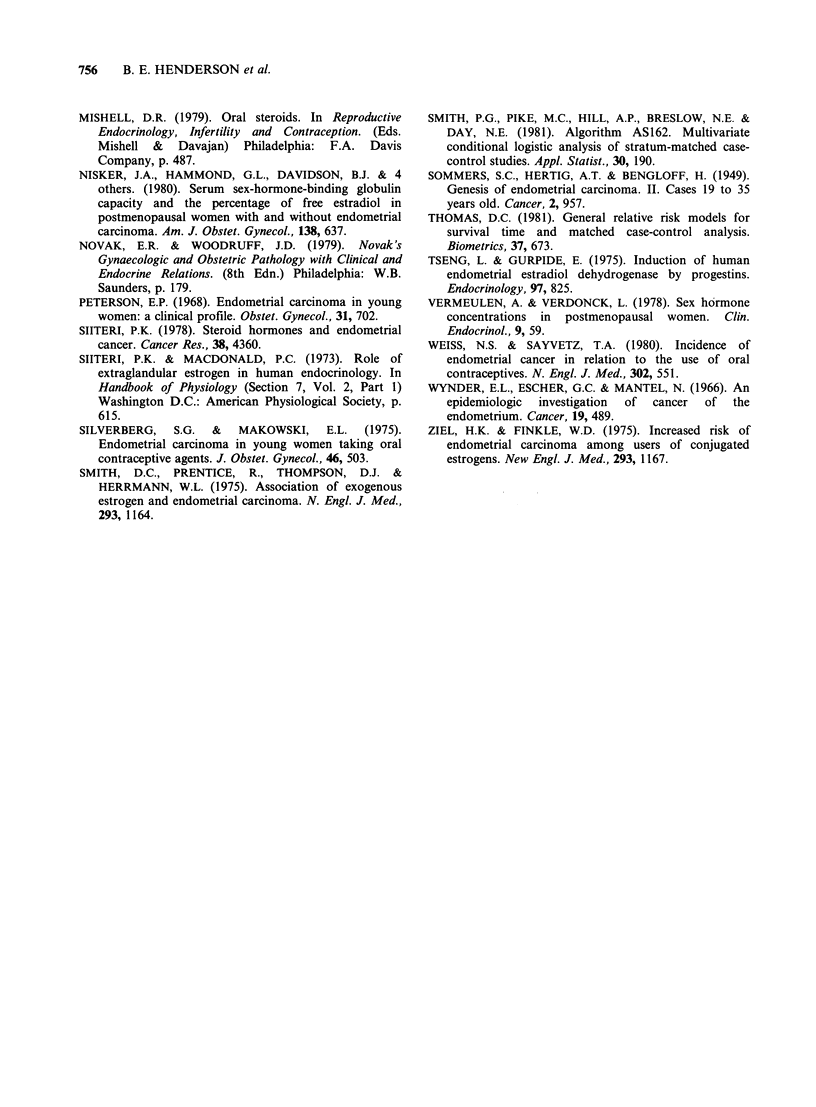# The epidemiology of endometrial cancer in young women.

**DOI:** 10.1038/bjc.1983.127

**Published:** 1983-06

**Authors:** B. E. Henderson, J. T. Casagrande, M. C. Pike, T. Mack, I. Rosario, A. Duke

## Abstract

A case-control study was conducted in Los Angeles County, California, of 127 endometrial cancer cases aged 45 years or less at diagnosis, to investigate the role of fertility, obesity and exogenous oestrogens in the development of the disease in young women. Use of sequential oral contraceptive (SOCs) or oestrogen replacement therapy (ERT) for greater than or equal to 2 years was strongly associated with increased risk of endometrial cancer. After excluding these cases, since the SOC or ERT use was probably the cause of their disease, we were left with 110 case-control pairs for further study. Among these remaining case-control pairs increasing parity was strongly associated with decreased risk (relative risk of 0.12 for women of parity 3 compared to nulliparous women, P less than 0.001). Current weight was associated with increased risk (relative risk of 17.7 for women weighing greater than or equal to 190 lbs compared to women weighing less than 130 lbs, P less than 0.001). Combination oral contraceptive (COC) use was associated with a decreased risk, which decreased with duration of COC use (relative risk of approximately 0.28 at 5 years of use, P less than 0.001), but the estimate of the protective effect was reduced and became statistically non-significant when allowance was made for weight and parity. The protective effect of COC use was only clearly evident in women who had less than 3 live-births and weighed less than 170 lbs. These results provide further support for the "unopposed" oestrogen hypothesis of the aetiology of endometrial cancer.


*Br. J. Cancer* (1983), **47**, 749–756

### The epidemiology of endometrial cancer in young women

B.E. Henderson, J.T. Casagrande, M.C. Pike, T. Mack, I. Rosario & A. Duke

*Department of Family and Preventive Medicine, University of Southern California School of Medicine, Los Angeles, California, U.S.A.*

**Summary** A case-control study was conducted in Los Angeles County, California, of 127 endometrial cancer cases aged 45 years or less at diagnosis, to investigate the role of fertility, obesity and exogenous oestrogens in the development of the disease in young women. Use of sequential oral contraceptive (SOCs) or oestrogen replacement therapy (ERT) for $\geq 2$ years was strongly associated with increased risk of endometrial cancer. After excluding these cases, since the SOC or ERT use was probably the cause of their disease, we were left with 110 case-control pairs for further study. Among these remaining case-control pairs increasing parity was strongly associated with decreased risk (relative risk of 0.12 for women of parity 3 compared to nulliparous women, $P<0.001$). Current weight was associated with increased risk (relative risk of 17.7 for women weighing $\geq 190$ lbs compared to women weighing $<130$ lbs, $P<0.001$). Combination oral contraceptive (COC) use was associated with a decreased risk, which decreased with duration of COC use (relative risk of $\sim 0.28$ at 5 years of use, $P<0.001$), but the estimate of the protective effect was reduced and became statistically non-significant when allowance was made for weight and parity. The protective effect of COC use was only clearly evident in women who had less than 3 live-births and weighed less than 170 lbs. These results provide further support for the "unopposed" oestrogen hypothesis of the aetiology of endometrial cancer.

Users of combination oral contraceptives have been reported to be at decreased risk of endometrial cancer in 4 recent case-control studies (Kaufman *et al.*, 1980; Weiss & Sayvetz, 1980; Hulka *et al.*, 1982; Kelsey *et al.*, 1982). It is not obvious how combination oral contraceptive use may "interact" with other endometrial cancer risk factors: in the above studies the numbers of ever-users of combination oral contraceptives were small, and the possible modifying effects of other risk factors were not (and probably could not be) discussed in any detail.

We report here the results of a case-control study of 127 endometrial cancer patients aged 45 years or less at diagnosis. By restricting attention to these young women we hoped to obtain a sufficient number of oral contraceptive users that the possible interaction with other risk factors could be evaluated.

### Methods

The cases were white women with microscopically confirmed adenocarcinoma of the endometrium (excluding carcinoma *in situ*) first diagnosed between January 1972 and December 1979. Any such woman, unless she had a Spanish surname and was born outside the United States, was eligible for inclusion if she was 45 years of age or less at diagnosis with no prior malignancies, was still alive and in the opinion of her physician was able to be interviewed, and was a resident of Los Angeles County at the time of her diagnosis. The cases were identified by the University of Southern California Cancer Surveillance Program (CSP), the population based cancer registry for Los Angeles County (Mack, 1977).

The CSP identified 185 such cases; 36 of these women refused to be interviewed. We obtained completed questionnaires on 149 (81%).

We sought one individually-matched control for each of these 149 cases. The control had to be white (excluding foreign-born if with a Spanish surname), have a birth date within 5 years of her matched case, have no prior malignancies, and have an intact uterus on her "pseudo-diagnosis" date (defined below). She also had to be at least as old at interview as her matched case was at diagnosis.

The controls were obtained by a procedure that defines a sequence of houses on specified neighbourhood blocks in the area where the matched case resided at the time of her diagnosis. Our goal was to interview the first matching female resident in the sequence. If no one was home at the time of visit, we left an explanatory letter and made 3 further follow-up visits some days later. In 110 instances, the first appropriate person agreed to participate. The second matched control cooperated in 14 instances, the third match in 2 instances, and the fourth match in 6 instances. For any patient, 80 housing units were visited before failure to secure a matched control was conceded. We were unable to locate a suitable match for 17 of the 149 cases. In all, 132 matched neighbourhood controls were

Correspondence: M.C. Pike

Received 5 December 1982; accepted 18 February 1983.

© The Macmillan Press Ltd., 1983

found and questionnaires completed. Five of the 132 sets were excluded from the analysis since the type of oral contraceptive (i.e. sequential or combination) used by one or other of the pair could not be determined. Such oral contraceptive information was lacking on 2 controls and on 3 cases, even after we contacted their physicians. This left a total of 127 case-control pairs.

One of us (I.R.) conducted all interviews by telephone. Information obtained included reproductive, menstrual, contraceptive, and gynaecological history, hormone and other drug use, and family history of cancer, up to the date of diagnosis of endometrial cancer. Each control was given a "pseudo-diagnosis" date which was the date on which she would have been the exact age her matched case was at her diagnosis of endometrial cancer. The diagnosis/pseudo-diagnosis date is referred to throughout the text as the "diagnosis date". The use of any drugs for the first time within 6 months of the diagnosis date was ignored.

The combination oral contraceptives commonly used in the U.S. contain either mestranol or ethinyl oestradiol as their oestrogenic component, and one of the 5 gestagens—norethindrone, norethynodrel, norethindrone acetate, ethynodiol diacetate or norgestrel. The relative oestrogenic potencies of the 2 oestrogens is $\sim 1$ and 2 respectively (Delforge & Ferin, 1970), and the relative potencies of the 5 gestagens is $\sim 1$, 1.09, 2, 15 and 30 respectively (Greenblatt, 1967). A potency ratio (E/G ratio) may be calculated (Mishell, 1979) for each preparation as (oestrogen component in mcg × relative potency) dividend by (gestagen component in mg × relative potency). The E/G ratio of the commonly-used preparations varies from 6.7 for Demulen to 175 for Ovcon-35 (Mishell, 1979). Although women frequently could not recall the precise oral contraceptive formulation used (a woman who took Ortho-Novum pills may not have remembered whether it was Ortho-Novum 1/50 or Ortho-Novum 1/80), the different preparations can be separated into a high E/G ratio group (E/G ratio over 45) and a low E/G ratio group such that more precise recall information was not needed.

Multivariate regression methods for individually matched case-control studies were used for statistical analysis (Breslow *et al.*, 1978; Holford *et al.*, 1978; Smith *et al.*, 1981; Thomas, 1981). The odds ratios estimated from such analyses closely approximate the related relative risks and the latter term is used in the text for clarity of presentation.

### Results

Eleven of the patients were aged $\leq 30$ years at diagnosis, 49 were aged 31–40, and 67 were aged 41–45. The socio-economic class of the patients and their individually-matched controls were very similar, as would be expected from the method of choosing controls by neighbourhood.

The relative risk (RR) for endometrial cancer was increased by long-term use of sequential oral contraceptives (SOCs) (Table I): 10 women had used SOCs for $\geq 2$ years, 9 of these women were cases. Five of the 9 cases with long-term SOC use had used C-Quens, 4 had used Oracon, and 1 each had used Norquens and Ortho-Novum SQ. As expected, oestrogen replacement therapy (ERT) was associated with increased risk (Table I), although the result was not statistically significant. One may conclude from these results and other published series (Lyon, 1975; Smith *et al.*, 1975; Ziel *et al.*, 1975; Mack *et al.*, 1976; McDonald *et al.*, 1977; Kelsey *et al.*, 1982) that long-term ($\geq 2$ years) SOC or ERT use was the cause of the endometrial cancer in most of the long-term SOC or ERT users. For this reason we excluded from all further analyses those case-control pairs in which either case or control used SOC or ERT for $>2$ years, leaving 110 case-control pairs (in 1 case-control pair both case and control had used SOC for $>2$ years).

**Table I** Relative risks for two known endometrial cancer risk factors

| *Factor* | | *Cases* | *Controls* | *RR** | P† |
|---|---|---|---|---|---|
| Sequential | 0 | 116 | 121 | 1.00 | 0.007‡ |
| O.C. (years) | <2 | 2 | 5 | 0.40 | |
| | 2+ | 9 | 1 | 4.60§ | |
| Oestrogen | 0 | 112 | 118 | 1.00 | 0.21‡ |
| replacement | <2 | 9 | 7 | 1.38 | |
| therapy | 2+ | 6 | 2 | 3.13 | |
| (years) | | | | | |

*Matched relative risk.
†1-sided statistical significance level.
‡For linear trend in logistic model to actual number of years of use.
§Estimated odds ratio at 3 years. Actual "matched" observed odds ratio is infinity, as the single control was matched to a case who took sequentials for more than 2 years.

The RR for endometrial cancer was significantly decreased by increasing numbers of full-term ($\geq 28$ weeks) pregnancies (FTPs) (Table II): a woman who had had $\geq 4$ FTPs had only 6% the risk of a nulliparous woman. Incomplete pregnancies (spontaneous and induced abortions) were associated with a slight decrease in risk (data not shown)—5.6 incomplete pregnancies was estimated

**Table II** Relative risks for various possible endometrial cancer risk factors

| *Factor* | | *Cases* | *Controls* | *RR** | P† |
|---|---|---|---|---|---|
| Full-term | 0 | 34 | 13 | 1.00 | <0.001‡ |
| pregnancies | 1 | 19 | 10 | 0.54 | |
| | 2 | 32 | 31 | 0.22 | |
| | 3 | 16 | 27 | 0.12 | |
| | 4+ | 9 | 29 | 0.06 | |
| Combination | 0 | 67 | 50 | 1.00 | <0.001§ |
| O.C. (years) | <2 | 23 | 22 | 0.75 | |
| | 2– | 12 | 11 | 0.79 | |
| | 4– | 4 | 9 | 0.28 | |
| | 6+ | 4 | 18 | 0.14 | |
| Current | –129 | 27 | 51 | 1.00 | <0.001¶ |
| weight | 130– | 28 | 38 | 1.45 | |
| (lbs) | 150– | 11 | 9 | 1.95 | |
| | 170– | 13 | 6 | 9.60 | |
| | 190+ | 31 | 6 | 17.70 | |
| Infertility‖ | No | 66 | 91 | 1.00 | <0.001 |
| | Yes | 44 | 19 | 3.50 | |
| Amenorrhea** | No | 92 | 105 | 1.00 | 0.001 |
| | Yes | 18 | 5 | 5.33 | |

*Matched relative risk.
†1-sided statistical significance level.
‡For linear trend in logistic model to actual number of live-births.
§For linear trend in logistic model to actual number of years of use.
¶For linear trend in logistic model to actual weight.
‖Unable to get pregnant for ≥3 years.
**Amenorrhea "requiring" visit to physician.

**Table III** Unmatched relative risks (RR) for combination O.C. (COC) use categorised by oestrogen to gestagen (E/G) ratio*

| | | | *Low E/G ratio COC use (years)* | | | |
|---|---|---|---|---|---|---|
| | | | 0 | <2 | 2– | 6+ |
| | 0 | Cases | 79 | 10 | 4 | 1 |
| | | Controls | 59 | 9 | 6 | 9 |
| | | RR | 1.00 | 0.83 | 0.50 | 0.08 |
| High | <2 | Cases | 5 | 1 | 1 | 1 |
| E/G | | Controls | 8 | 2 | 2 | 0 |
| ratio | | RR | 0.47 | 0.37 | 0.37 | 0.00 |
| COC | 2– | Cases | 4 | 3 | 0 | 0 |
| use | | Controls | 7 | 0 | 2 | 0 |
| (years) | | RR | 0.43 | 0.00 | 0.00 | — |
| | 6+ | Cases | 1 | 0 | 0 | 0 |
| | | Controls | 5 | 1 | 0 | 0 |
| | | RR | 0.15 | 0.00 | — | — |

*See Methods section for definition of E/G ratio.

as equivalent to 1 FTP in terms of risk reduction—but this decrease was not statistically significant (1-sided $P=0.31$) and we do not consider incomplete pregnancies further in this analysis. Age at first FTP was not associated with any change in risk when considered jointly with numbers of FTPs (1-sided $P=0.49$). Similarly age at last FTP was not associated with any change in risk when considered jointly with numbers of FTPs (1-sided $P=0.20$). The RRs associated with numbers of FTPs were not significantly affected by age at first FTP or age at last FTP.

The RR for endometrial cancer decreased steadily with increasing use of combination oral contraceptives (COCs): a woman with ≥6 years of COC use had 14% the risk of a woman who never used COCs (Table II). Recent COC use (i.e. use within 1 year of the diagnosis date) was positively associated with duration of COC use. However, there was no effect of recency of COC use over and above the effect explicable by this association with duration of COC use. Table III shows that use of COCs with either "High" or "Low" oestrogen to gestagen ratios was associated with a reduced risk of endometrial cancer, and the effect was not clearly dependent on the E/G ratio at least over the range defined by our crude division into "High" and "Low".

The RR for endometrial cancer was significantly increased by increasing "current" weight (i.e. weight recorded 1 year prior to diagnosis): a woman who weighed ≥190lbs had almost 18 times the risk of a woman who weighed <130lbs. (Table II). Quetelet's index (weight/height$^2$) was also significantly positively related to the risk of endometrial cancer. In this data, weight and Quetelet's index were very highly correlated, so that neither relationship with endometrial cancer risk was significant after allowance was made for the other, and Quetelet's index was slightly less discriminatory than weight alone. We have therefore presented only the results for weight.

Weight at age 18 was strongly associated with risk of endometrial cancer (see second last line in Table IV). The effect could, however, be almost completely "explained" by current weight (see last line in Table IV), so that weight at age 18 does not add much further information if current weight is known. The RR associated with current weight was only slightly modified by knowledge of weight at age 18. Five cases but no controls had been diagnosed as having diabetes. The cases weighed 185, 195, 200, 225 and 250lbs so that the increased risk cannot be distinguished from that due to weight alone.

Long-term infertility, defined as a "recognised" inability to get pregnant for ≥3 years, was

**Table IV** Unmatched relative risks (RR) for weight at age 18 stratified by current weight

| Current weight | | Weight at age 18 | | |
|---|---|---|---|---|
| | | −129 | 130− | 170+ |
| −129 | Cases | 23 | 4 | 0 |
| | Controls | 48 | 3 | 0 |
| | RR | 1.00 | 2.78 | — |
| 130− | Cases | 22 | 16 | 1 |
| | Controls | 28 | 18 | 1 |
| | RR | 1.00 | 1.13 | 1.27 |
| 170+ | Cases | 14 | 24 | 6 |
| | Controls | 3 | 8 | 1 |
| | RR | 1.00 | 0.64 | 1.29 |
| All | Cases | 59 | 44 | 7 |
| | Controls | 79 | 29 | 2 |
| | RR | 1.00 | 2.10 | 4.75 |
| RR adjusted for current weight | | 1.00 | 1.17 | 1.28 |

associated with a 3.5-fold increased risk of endometrial cancer (Table II). Infertility of shorter duration was not associated with an increased risk; and there was no further increase in risk with recognised infertility of longer duration. Amenorrhea "requiring" a physician visit was associated with a 5.3-fold increased risk of endometrial cancer (Table II).

Table V shows the maximum likelihood estimates of the logistic parameters (log RRs) for each of the 5 risk factors in Table II fitted singly. All were statistically significant. The Table also shows the estimated parameters and associated significance levels when the 5 factors were fitted together. Only FTPs, current weight and amenorrhea remained statistically significant at the conventional 1-sided 5% level.

**Table V** Logistic analysis of various possible endometrial cancer risk factors

| Factor | Fitted singly* $\hat{b}$ | RR | P | Fitted together*† $\hat{b}$ | RR | P |
|---|---|---|---|---|---|---|
| Full-term pregnancies | −0.61 | 0.54 | <0.001 | −0.51 | 0.61 | <0.001 |
| Combination O.C. (1 year increments) | −0.17 | 0.85 | <0.001 | −0.058 | 0.94 | 0.17 |
| Current weight (10 lb increments) | 0.23 | 1.26 | <0.001 | 0.17 | 1.19 | <0.001 |
| Infertility | 1.25 | 3.50 | <0.001 | 0.71 | 2.02 | 0.07 |
| Amenorrhea | 1.67 | 5.33 | <0.001 | 1.97 | 7.19 | 0.005 |

*1-sided statistical significance level.
†After fitting other 4 factors.

The matched RRs associated with current weight ≥170 lbs were very large, and since it was possible that this extreme level of risk was dominating the multivariate analysis and making it difficult to evaluate the effects of other potential risk factors, we looked at the other risk factors stratified by weight (Table VI).

**Table VI** Unmatched relative risks (RR) for various possible endometrial cancer risk factors stratified by current weight

| Current weight | | Full-term pregnancies | | | | |
|---|---|---|---|---|---|---|
| | | 0 | 1 | 2 | 3 | 4+ |
| −169 | Cases | 23 | 7 | 17 | 11 | 8 |
| | Controls | 12 | 9 | 30 | 21 | 26 |
| | RR | 1.00 | 0.41 | 0.30 | 0.27 | 0.16 |
| 170+ | Cases | 11 | 12 | 15 | 5 | 1 |
| | Controls | 1 | 1 | 1 | 6 | 3 |
| | RR | 1.00 | 1.09 | 1.36 | 0.08 | 0.03 |
| | | *Combination O.C. (years)* | | | | |
| | | 0 | <2 | 2− | 4− | 6+ |
| −169 | Cases | 41 | 13 | 7 | 2 | 3 |
| | Controls | 41 | 21 | 10 | 9 | 17 |
| | RR | 1.00 | 0.62 | 0.70 | 0.22 | 0.18 |
| 170+ | Cases | 26 | 10 | 5 | 2 | 1 |
| | Controls | 9 | 1 | 1 | 0 | 1 |
| | RR | 1.00 | 3.46 | 1.73 | 1.04 | |
| | | *Infertility* | | *Amenorrhea* | | |
| | | N | Y | N | Y | |
| −169 | Cases | 46 | 20 | 64 | 2 | |
| | Controls | 80 | 18 | 93 | 5 | |
| | RR | 1.00 | 1.93 | 1.00 | 0.58 | |
| 170+ | Cases | 20 | 24 | 28 | 16 | |
| | Controls | 11 | 1 | 12 | 0 | |
| | RR | 1.00 | 13.20 | 1.00 | ∞ | |

The protective effect of increasing numbers of FTPs was evident both in the "obese" women (current weight ≥170 lbs) and in the "non-obese" women. The protective effect of increasing COC use was evident only in the non-obese women. Infertility was associated with a larger increase in risk in obese women (unmatched RR = 13.20), but was also evident in the non-obese women (unmatched RR = 1.93). The increased risk associated with amenorrhea was also greatly elevated in obese women, and there was no increased risk in the non-obese.

Table VII shows the matched RRs for the 5 risk factors of interest for the 63 case-control pairs in which both members weighed <170 lbs. The RRs

**Table VII** Relative risks for various possible endometrial cancer risk factors: 63 case-control pairs with weight <170 lbs

| *Factor* | | *Cases* | *Controls* | *RR** | P† |
|---|---|---|---|---|---|
| Full-term | 0 | 21 | 6 | 1.00 | 0.001‡ |
| pregnancies | 1 | 7 | 7 | 0.13 | |
| | 2 | 16 | 17 | 0.15 | |
| | 3 | 11 | 14 | 0.12 | |
| | 4+ | 8 | 19 | 0.05 | |
| Combination | 0 | 39 | 27 | 1.00 | 0.01§ |
| O.C. (years) | <2 | 13 | 14 | 0.68 | |
| | 2– | 6 | 6 | 0.89 | |
| | 4– | 2 | 6 | 0.33 | |
| | 6+ | 3 | 10 | 0.20 | |
| Current | –129 | 27 | 33 | 1.00 | 0.16¶ |
| weight (lbs) | 130– | 26 | 23 | 1.30 | |
| | 150–169 | 10 | 7 | 1.57 | |
| Infertility | No | 43 | 52 | 1.00 | 0.03 |
| | Yes | 20 | 11 | 2.29 | |
| Amenorrhea | No | 61 | 63 | 1.00 | 0.25 |
| | Yes | 2 | 0 | ∞ | |

*Matched relative risk.
†1-sided statistical significance level.
‡For linear trend in logistic model to actual number of live-births.
§For linear trend in logistic model to actual number of years of use.
¶For linear trend in logistic model to actual weight.

are all consistent with those shown for all women in Table II. Table VIII shows the multivariate analysis for the 3 statistically significant risk factors (FTPs, COC and infertility): only numbers of FTPs remained statistically significant when the factors were considered jointly. One FTP was the equivalent of ~5 years of COC use (0.41/0.080). The reason for the disappearance of infertility as a risk factor was that 50% (10/20) of the infertile cases were nulliparous, while only 18% (2/11) of such controls were nulliparous.

**Table VIII** Logistic analysis of various possible endometrial cancer risk factors: 63 case-control pairs with weight <170 lbs

| | *Fitted singly* | | | *Fitted together* | | |
|---|---|---|---|---|---|---|
| *Factor* | $\hat{b}$ | *RR* | P* | $\hat{b}$ | *RR* | P*† |
| Full-term pregnancies | –0.46 | 0.63 | 0.001 | –0.41 | 0.66 | 0.001 |
| Combination O.C. (1 year increments) | –0.13 | 0.88 | 0.01 | –0.080 | 0.92 | 0.10 |
| Infertility | 0.83 | 2.29 | 0.03 | 0.08 | 1.08 | 0.44 |

*1-sided statistical significance level.
†After fitting other 2 factors.

There was no effect of age at menarche or age at establishment of regular cycles (or duration of time from menarche to establishment of regular cycles) on the risk of endometrial cancer.

There was no evidence of any increased risk associated with intra-uterine contraceptive devices.

The stage of the endometrial cancer at diagnosis could be determined from the CSP records in 72/110 cases. Forty-three had strictly localised disease (local) and 29 had tumour extension at least into the distal two thirds of the myometrium (invasive). The above risk factors were clearly evident for both groups, but the number of invasive cases was not sufficient to be sure that the magnitudes of the effects were the same.

One case and 1 control reported that they had been diagnosed with polycystic ovarian disease (PCO) before the diagnosis date. Three additional cases were found to have PCO at the time of diagnosis. The PCO cases weighed 130, 175, 200 and 220 lbs, and all were infertile.

Five cases, but only 1 control, reported endometrial cancer in a first degree relative (cases—2 mothers and 3 sisters; controls—1 mother). An additional 5 cases and 1 control reported cancer of the cervix in a first degree relative; some of these may actually have been carcinomas of the endometrium. Breast cancer in a first degree relative was reported by 4 cases and 7 controls.

### Discussion

The present case-control study of young white women in the Los Angeles area shows clearly that long-term use of sequential oral contraceptives (SOCs) increases the risk of endometrial cancer. SOCs were removed from sale in 1976 because of case reports of endometrial cancer in SOC users (Lyon, 1975), and data suggesting that among young endometrial cancer cases who took oral contraceptives the proportion taking SOCs was disproportionately high (Silverberg & Makowski, 1975). The oestrogenic component of Oracon is 0.1 mg ethinyl oestradiol while the oestrogenic components of the other SOCs is 0.08 mg mestranol; mestranol has only half the oestrogenic potency of ethinyl oestradiol (Greenblatt, 1960). This suggests that Oracon may be more strongly related to endometrial cancer. In the study of Weiss & Sayvetz (1980) the increased risk of SOCs appeared to be restricted to Oracon users, but we found no evidence of an especially raised risk with this formulation (SOC users: Oracon—6 cases and 3 controls; C-Quens—5 cases and 3 controls; other formulations—2 cases and 1 control). We do not have enough data to settle this question. Only 3/9

long-term SOC cases used SOCs within 6 months of their date of diagnosis, and it therefore appears that the increased risk is not restricted to current users.

Although the increased risk associated with oestrogen replacement therapy (ERT) was not statistically significant, the magnitude of the observed risk was compatible with that found in many studies of post-menopausal endometrial cancer cases (Smith *et al.*, 1975; Ziel *et al.*, 1975; Mack *et al.*, 1976; McDonald *et al.*, 1977; Kelsey *et al.*, 1982).

We eliminated from all further analyses the 17 case-control pairs in which either the case or the control had used SOCs or ERT for $\geq 2$ years. We did this in order to simplify the presentation and so that we did not need to build these 2 variables into all calculations of relative risks for other factors. The usual straightforward "adjustment" using logistic methods assumes that the relative risks of different factors is multiplicative and we saw no reason to assume this for the sake of retaining these 17 pairs.

The present study provides clear evidence that the risk of endometrial cancer in young women is markedly increased by obesity. It is well known that obesity is a major risk factor for endometrial cancer in post-menopausal women (Damon, 1960; Wynder *et al.*, 1966; Elwood *et al.*, 1977; Kelsey *et al.*, 1982; La Vecchia *et al.*, 1982), and case reports have suggested that young endometrial cancer cases tend to be very obese (Sommers *et al.*, 1949; Dockerty *et al.*, 1951; Peterson, 1968). In our population 50% (55/110) of the cases weighed $\geq 150$ lbs, and the associated attributable risk percent (ARP) is 38%, i.e. 38% of the current cases of endometrial cancer in Los Angeles County in young women are attributable to obesity, defined as weighing $\geq 150$ lbs.

Obesity is thus now clearly established as an extremely important (and presumably preventable) cause of endometrial cancer both in pre-menopausal and post-menopausal women. Table VI shows that if the obesity is associated with infertility or amenorrhea the risk of endometrial cancer is particularly high.

This study shows that the risk of endometrial cancer in young women is reduced considerably by increasing numbers of FTPs. This is consistent with the results of studies of older mainly post-menopausal women (Damon, 1960; Wynder *et al.*, 1966; Elwood *et al.*, 1977; Kelsey *et al.*, 1982; La Vecchia *et al.*, 1982). Incomplete pregnancies also appear to be protective. The extent of protection afforded by an incomplete pregnancy relative to that afforded by a FTP is roughly in proportion to the length of the pregnancy. The decreased risk associated with FTPs was not due to any relationship between weight and numbers of FTPs. The results shown in Tables II and VII suggest a larger protective effect from first than from subsequent pregnancies. Although analysis showed that this effect was not statistically significant, such an effect should be looked for in future studies.

Although the current study has only 1 less endometrial cancer case who had ever used COCs than all other studies combined (Kaufman *et al.*, 1980; Weiss & Sayvetz, 1980; Hulka *et al.*, 1982; Kelsey *et al.*, 1982), we still have too little data to be able to accurately estimate the protective effect of COC use or to confidently delineate the group of women for whom it is protective. We saw in Table VI that COC use does not appear to be protective in obese women, and the decreased risk in non-obese women is not statistically significant when considered together with numbers of FTPs. Inspection of the data (not shown) suggests that the reason for the latter reduction in effect is that COC use does not appear to be protective in women with $>3$ FTPs. Further study of large numbers of young endometrial cancer cases is needed to settle these issues.

The greatly increased risk associated with obesity in post-menopausal women has been interpreted in "excess" oestrogen terms by a number of authors (Siiteri, 1978; Nisker *et al.*, 1980; Henderson *et al.*, 1982). Plasma oestrogen in the post-menopausal woman is largely derived by extra-glandular conversion of androstenedione to oestrone, and the rate of this aromatization increases with body weight since adipose tissue is particularly rich in the necessary enzymes (Siiteri & MacDonald, 1973). Oestradiol is then derived from peripheral conversion of oestrone (Vermeulen & Verdonck, 1978). The bio-availability of oestradiol may be related to the concentration of sex-hormone-binding globulin (SHBG) in the serum, and recent results show that the SHBG concentration is lower in obese women (Nisker *et al.*, 1980). Thus, obese women not only have greater concentrations of circulating oestrogens, but the oestradiol may be more available to oestrogen-responsive tissue.

Progesterone and other gestagens have profound effects on the endometrium and are therapeutically useful in treating both endometrial hyperplasia and carcinoma. Gestagens increase the activity of the dehydrogenase that converts oestradiol to the biologically less active oestrone (Tseng & Gurpide, 1976); and gestagens decrease the concentration of oestradiol receptors (Hsueh *et al.*, 1975). Maximum mitotic activity in the endometrium occurs in the follicular phase of the cycle, and gestagens cause differentiation of the endometrial cells to a secretory state (Novak & Woodruff, 1979).

The "unopposed oestrogen hypothesis" for endometrial cancer joins these two factors together (Siiteri, 1978; Nisker *et al.*, 1980; Henderson *et al.*, 1982). Our results showing the especially increased rate of endometrial cancer in obese women with amenorrhea provide very strong support for this hypothesis. Increased fertility may protect against endometrial cancer directly—the state of the endometrium during pregnancy being very similar to its state during the luteal phase of the cycle with little mitotic activity. Part of the protection from increased parity may, however, be indirect—high parity may imply a low frequency of anovular cycles in which little or no progesterone is produced.

Protection from long-term COC use also supports the unopposed oestrogen hypothesis—the gestagen component of COCs normally being sufficiently potent to prevent proliferation of the endometrium (Anderson, 1979). The dose of gestagen may, however, not be sufficiently strong to inhibit endometrial proliferation in very obese women; this would explain our failure to find a protective effect of COC use in such women. We have not been able to find information about the state of the endometrium in very obese women on COCs.

Although there was no overall effect of age at menarche or duration of time from menarche to establishment of regular cycles on endometrial cancer risk, these negative results appeared to be due to essentially contradictory results in different weight categories of cases. Relative to controls the obese cases tended to have early menarche and long duration to establishment of regular cycles, while the non-obese cases tended to have late menarche and short duration to establishment of regular cycles. None of these results were, however, statistically significant and a large study will be neede to shed light on this question.

We wish to thank all the patients and controls who participated in this study—their willingness to answer both our initial and follow-up questions is deeply appreciated.

We are most grateful to Mrs. J. Howland for superb secretarial assistance throughout this study.

This work was supported by grants (CA 17054 and CA 14089) from the National Cancer Institute, National Institute of Health, U.S. Public Health Service.

### References

ANDERSON, M.C. (1979). *Syntax Atlas of Gynaecology*. London: Hamblin Learning Systems Limited.

BRESLOW, N.E., DAY, N.E., HALVORSEN, K.T., PRENTICE, R.L. & SABAI, C. (1978). Estimation of multiple relative risk functions in matched case-control studies. *Am. J. Epidemiol.*, **108**, 299.

DAMON, A. (1960). Host factors in cancer of the breast and uterine cervix and corpus. *J. Natl Cancer Inst.*, **24**, 483.

DELFORGE, J.P. & FERRIN, J. (1970). A histometric study of two estrogens: ethinyl estradiol and its 3-methyl-ether derivative (mestranol); their comparative effect upon the growth of the human endometrium. *Contraception*, **1**, 57.

DOCKERTY, M.B., LOVELADY, S.B. & FOUST, G.T. (1951). Carcinoma of the corpus uteri in young women. *Am. J. Obstet. Gynecol.*, **61**, 966.

ELWOOD, J.M., COLE, P., ROTHMAN, K.J. & KAPLAN, S.D. (1977). Epidemiology of endometrial cancer. *J. Natl Cancer Inst.*, **59**, 1055.

GREENBLATT, R.B. (1967). Progestational agents in clinical practice. *Med. Sci.*, **18**, 37.

HENDERSON, B.E., ROSS, R.K., PIKE, M.C. & CASAGRANDE, J.T. (1982). Endogenous hormones as a major factor in human cancer. *Cancer Res.*, **42**, 3232.

HOLFORD, T.R., WHITE, C. & KELSEY, J.L. (1978). Multivariate analysis for matched case-control studies. *Am. J. Epidemiol.*, **107**, 245.

HULKA, B.S., CHAMBLISS, L.E., KAUFMAN, D.G., FOWLER, W.C. & GREENBERG, B.G. (1982). Protection against endometrial carcinoma by combination-product oral contraceptives. *J.A.M.A.*, **247**, 475.

HSUEH, A.J.W., PECK, E.J. & CLARK, J.H. (1975). Progesterone antagonism of the oestrogen receptor and oestrogen-induced uterine growth. *Nature*, **254**, 337.

KAUFMAN, D.W., SHAPIRO, S., SLONE, D. & 10 others. (1980). Decreased risk of endometrial cancer among oral contraceptive users. *N. Engl. J. Med.*, **303**, 1045.

KELSEY, J.L., LI VOLSI, V.A., HOLFORD, T.R. & 5 others. (1982). A case-control study of cancer of the endometrium. *Am. J. Epidemiol.*, **116**, 333.

LA VECCHIA, C., FRANCISCHI, S., GALLUS, G. & 4 others. (1982). Oestrogens and obesity as risk factors for endometrial cancer in Italy. *Int. J. Epidemiol.*, **11**, 120.

LYON, F.A. (1975). The development of adenocarcinoma of the endometrium in young women receiving long-term sequential oral contraception. *Am. J. Obstet. Gynecol.*, **123**, 299.

MACK, T. (1977). Cancer Surveillance Program in Los Angeles County. *Natl Cancer Inst. Monogr.*, **47**, 99.

MACK, T.M., PIKE, M.C., HENDERSON, B.E., PFEFFER, R.I., GERKINS, V.R. & ARTHUR, M. (1976). Estrogens and endometrial cancer in a retirement community. *N. Engl. J. Med.*, **294**, 1262.

McDONALD, T.W., ANNEGERS, J.F., FALLON, W.M. & 3 others (1977). Exogenous estrogens and endometrial carcinoma: case-control and incidence study. *Am. J. Obstet. Gynecol.*, **127**, 572.

MEISSNER, W.A., SOMMERS, S.C. & SHERMAN, B.M. (1957). Endometrial hyperplasia, endometrial carcinoma, and endometriosis produced experimentally by estrogen. *Cancer*, **10**, 500.

MISHELL, D.R. (1979). Oral steroids. In *Reproductive Endocrinology, Infertility and Contraception.* (Eds. Mishell & Davajan) Philadelphia: F.A. Davis Company, p. 487.

NISKER, J.A., HAMMOND, G.L., DAVIDSON, B.J. & 4 others. (1980). Serum sex-hormone-binding globulin capacity and the percentage of free estradiol in postmenopausal women with and without endometrial carcinoma. *Am. J. Obstet. Gynecol.*, **138**, 637.

NOVAK, E.R. & WOODRUFF, J.D. (1979). *Novak's Gynaecologic and Obstetric Pathology with Clinical and Endocrine Relations.* (8th Edn.) Philadelphia: W.B. Saunders, p. 179.

PETERSON, E.P. (1968). Endometrial carcinoma in young women: a clinical profile. *Obstet. Gynecol.*, **31**, 702.

SIITERI, P.K. (1978). Steroid hormones and endometrial cancer. *Cancer Res.*, **38**, 4360.

SIITERI, P.K. & MACDONALD, P.C. (1973). Role of extraglandular estrogen in human endocrinology. In *Handbook of Physiology* (Section 7, Vol. 2, Part 1) Washington D.C.: American Physiological Society, p. 615.

SILVERBERG, S.G. & MAKOWSKI, E.L. (1975). Endometrial carcinoma in young women taking oral contraceptive agents. *J. Obstet. Gynecol.*, **46**, 503.

SMITH, D.C., PRENTICE, R., THOMPSON, D.J. & HERRMANN, W.L. (1975). Association of exogenous estrogen and endometrial carcinoma. *N. Engl. J. Med.*, **293**, 1164.

SMITH, P.G., PIKE, M.C., HILL, A.P., BRESLOW, N.E. & DAY, N.E. (1981). Algorithm AS162. Multivariate conditional logistic analysis of stratum-matched case-control studies. *Appl. Statist.*, **30**, 190.

SOMMERS, S.C., HERTIG, A.T. & BENGLOFF, H. (1949). Genesis of endometrial carcinoma. II. Cases 19 to 35 years old. *Cancer*, **2**, 957.

THOMAS, D.C. (1981). General relative risk models for survival time and matched case-control analysis. *Biometrics*, **37**, 673.

TSENG, L. & GURPIDE, E. (1975). Induction of human endometrial estradiol dehydrogenase by progestins. *Endocrinology*, **97**, 825.

VERMEULEN, A. & VERDONCK, L. (1978). Sex hormone concentrations in postmenopausal women. *Clin. Endocrinol.*, **9**, 59.

WEISS, N.S. & SAYVETZ, T.A. (1980). Incidence of endometrial cancer in relation to the use of oral contraceptives. *N. Engl. J. Med.*, **302**, 551.

WYNDER, E.L., ESCHER, G.C. & MANTEL, N. (1966). An epidemiologic investigation of cancer of the endometrium. *Cancer*, **19**, 489.

ZIEL, H.K. & FINKLE, W.D. (1975). Increased risk of endometrial carcinoma among users of conjugated estrogens. *New Engl. J. Med.*, **293**, 1167.